# Selected solid-state behaviour of three di-*tert*-butyl-substituted *N*-salicylideneaniline derivatives: temperature-induced phase transitions and chromic behaviour

**DOI:** 10.1107/S2053229621008780

**Published:** 2021-09-29

**Authors:** Helen E. Mason, Judith A. K. Howard, Hazel A. Sparkes

**Affiliations:** a Department of Chemistry, Durham University, South Road, Durham DH1 3LE, UK; b School of Chemistry, University of Bristol, Cantock’s Close, Bristol BS8 1TS, UK

**Keywords:** Schiff base, chromism, polymorphism, phase transition, crystal structure

## Abstract

The synthesis, single-crystal structures and chromic behaviour of three related Schiff bases, namely, (*E*)-2,4-di-*tert*-butyl-6-{[(4-fluoro­phen­yl)imino]­meth­yl}phenol, C_21_H_26_FNO, **1**, (*E*)-2,4-di-*tert*-butyl-6-{[(4-chloro­phen­yl)imino]­meth­yl}phenol, C_21_H_26_ClNO, **2**, and (*E*)-6-{[(4-bromo­phen­yl)imino]­meth­yl}-2,4-di-*tert*-butyl­phenol, C_21_H_26_BrNO, **3**, are reported. Two polymorphs of **1** were obtained, which were found to have different photochromic properties. Schiff bases **2** and **3** were found to be isostructural and underwent a phase transition upon cooling which was attributed to the dynamic disorder in one of the *tert*-butyl groups resolving at low temperature. All of the structures were found to exist in the enol rather than the keto form based on the C—O(H) and imine C=N bond lengths, and contained an intra­molecular O—H⋯N hydrogen bond alongside weaker inter­molecular C—H⋯O contacts.

## Abstract 2

Three di-*tert*-butyl-substituted *N*-salicylideneaniline derivatives were synthesized, structurally characterized and their chromic properties investigated. Polymorphism was found to affect the photochromic behaviour of the first com­pound, while the second two com­pounds were found to display a temperature-induced phase transition. All of the structures contained an intra­molecular O—H⋯N hydrogen bond.

## Introduction   

Compounds which display reversible property changes upon some sort of stimulus are of inter­est due to potential applications, including optical switches (Sliwa *et al.*, 2005 ▸), sensors (Sahu *et al.*, 2020 ▸) or optical data storage (Wang *et al.*, 2020 ▸). Within these are com­pounds displaying temperature-dependent thermochromic (Seeboth *et al.*, 2014 ▸; Suzuki *et al.*, 2019 ▸) or light-induced photochromic (Wu *et al.*, 2020 ▸) colour changes. One such class of com­pounds that has been found to exhibit both thermochromism and photochromism in the solid state are *N*-salicylideneanilines, Schiff bases of salicyl­aldehyde derivatives with aniline derivatives (Senier & Shepheard, 1909 ▸; Cohen & Schmidt, 1962 ▸; Cohen *et al.*, 1964 ▸). Typically, their thermochromism involves a lightening of colour from red/orange to orange/yellow upon cooling, while their photochromic colour changes usually result in a darkening of colour from yellow to orange/red upon irradiation with UV light. Initially, the two properties were thought to be mutually exclusive (Cohen & Schmidt, 1962 ▸; Cohen *et al.*, 1964 ▸); how­ever, now it is believed that most, if not all, of *N*-sali­cyl­idene­anilines display thermochromism, with some also showing photochromism (Fujiwara *et al.*, 2004 ▸).

**Figure 1:**
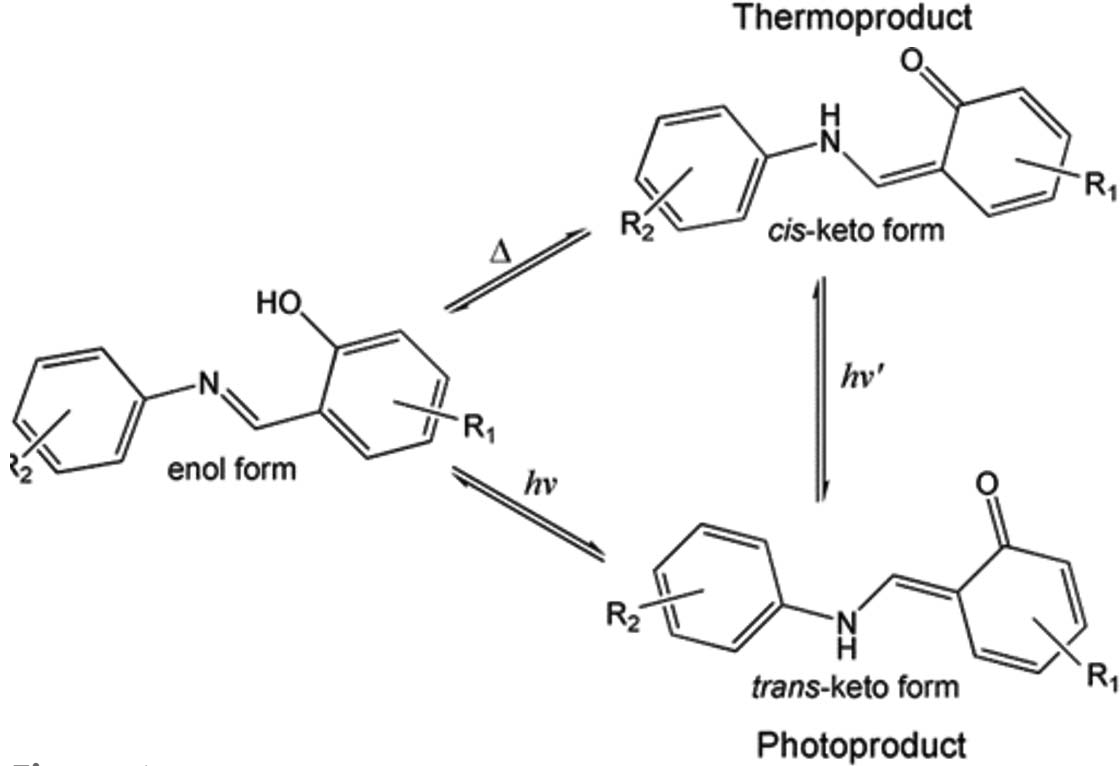
Illustration of the proposed mechanism for the thermo- or photochromism in *N*-salicylideneaniline derivatives.

The mechanism for the thermochromic colour change is believed to be due to an enol to *cis*-keto tautomerism, while the photochromism involves a *cis* to *trans* isomerism of the keto form (Hadjoudis & Mavridis, 2004 ▸; Robert *et al.*, 2009 ▸) (Fig. 1 ▸). Evidence of the thermoproduct has been observed for *N*-(5-chloro­salicyl­idene)-4-hy­droxy­aniline, where the population of the *cis*-keto form was found to increase upon cooling (Ogawa *et al.*, 1998 ▸, 2000 ▸), while the photoproduct has been seen crystallographically for *N*-3,5-di-*tert*-butyl­salicyl­idene-3-nitro­aniline using two-photon irradiation (Harada *et al.*, 1999 ▸). The enol form is believed to be colourless, while the keto form is coloured (Ogawa *et al.*, 1998 ▸; Fujiwara *et al.*, 2004 ▸; Harada *et al.*, 2007 ▸). However, the thermochromism cannot be fully explained by the keto–enol tautomerism alone. In order to fully explain the thermochromism, it is necessary to take into account fluorescence (Harada *et al.*, 2007 ▸). The impact of fluorescence is particularly significant for thermochromic com­pounds at lower temperature and can in fact be the dominant cause of colour change, while at higher temperatures, fluorescence is negligible. The presence of fluorescence at lower temperature results in different perceived colours to those observed from the diffuse reflectance spectra, *e.g. N*-(5-chloro-2-hy­droxy­benzyl­idene)aniline appears yellowish green at 80 K but diffuse reflectance suggests the colour to be pale yellow since fluorescence was eliminated in the measurement of diffuse reflectance spectra. The extent of the thermochromism of the *N*-salicylideneanilines has been linked to the dihedral angle (Φ) between the two aromatic rings, with those having Φ < 25° being generally strongly thermochromic, as a smaller inter­planar or dihedral angle results in reduced over­lap between the N-atom lone pair and the aromatic aniline moiety. This allows for easier H-atom transfer and creates a stronger intra­molecular hydrogen bond. While a larger dihedral angle increases overlap between the N-atom lone pair and the aromatic aniline moiety giving greater delocalization into the π-system and reducing the basicity of the N atom and thus the thermochromism (Hadjoudis & Mavridis, 2004 ▸; Robert *et al.*, 2009 ▸). For photochromism, the link to dihedral angle is more com­plicated and com­pounds with Φ < 20° are generally nonphotochromic, those with Φ > 30° are more likely to be photochromic and those in between can be either photochromic or nonphotochromic (Johmoto *et al.*, 2012 ▸). Other factors have also been found to influence the chromic behaviour of the *N*-salicylideneanilines, including substituents that weaken the O—H bond or increase the basicity of the N atoms, tending to result in more strongly thermochromic com­pounds (Hadjoudis & Mavridis, 2004 ▸). In addition, crystal packing also affects chromic behaviour, with more closely packed structures tending to be more strongly thermochromic and more open packed structures more likely to be photochromic (Hadjoudis & Mavridis, 2004 ▸; Robert *et al.*, 2009 ▸). The latter is likely to be due to the large conformational change required for the transition, with tightly packed structures having greater steric restrictions to conformational change. The presence of bulky groups, such as *tert*-butyl substituents, or creating cavities using hosts can help to increase space in the lattice of a structure and favour photochromism, as more space presumably reduces the steric restraint on the mol­ecule for the significant conformational change required for *cis* to *trans* isomerism to occur (Johmoto *et al.*, 2012 ▸, Pistolis *et al.*, 1996 ▸; Koyama *et al.*, 1994 ▸).
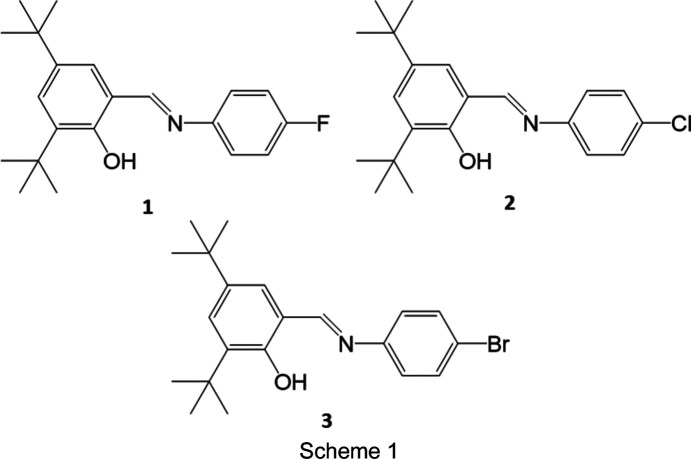


The structures of three related *N*-salicylidineanlines, namely, (*E*)-2,4-di-*tert*-butyl-6-{[(4-fluoro­phen­yl)imino]­meth­yl}phenol, **1**, (*E*)-2,4-di-*tert*-butyl-6-{[(4-chloro­phen­yl)imino]­meth­yl}phenol, **2**, and (*E*)-6-{[(4-bromo­phen­yl)imino]­meth­yl}-2,4-di-*tert*-butyl­phenol, **3**, are reported alongside a study into their chromic properties (Scheme 1). The structure of **2** has been reported previously at 273 K (Li *et al.*, 2007 ▸), but no investigation into the chromic properties or thermal behaviour has been reported. The structure of **3** has also been reported and is known to be photochromic; however, the thermal behaviour was not studied (Johmoto *et al.*, 2012 ▸).

## Experimental   

### Synthesis   

All reagents were used as supplied by Aldrich. Compounds were synthesized by direct condensation of the appropriate salicyl­aldehyde and aniline derivatives in ethanol. 1.25 (for **1** and **3**) or 2.5 mmoles (for **2**) of the salicyl­aldehyde and aniline were each dissolved in ethanol (25 ml), and the resulting solutions combined and refluxed with stirring for 4 h. Any precipitate was filtered off, rinsed with ethanol and left to dry. The (remaining) solution was then removed under reduced pressure using a rotary evaporator until (further) precipitate formed. Recrystallization was carried out by slow evaporation from ethanol.

### Characterization   

Elemental C, H and N content analysis was carried out by the Durham University Analytical service using an Exeter Analytical E-440 Elemental Analyzer.

### Single-crystal X-ray diffraction data collection and refinement   

Details of the X-ray data collection and refinement are provided in Table 1 ▸ and Table S1 in the supporting information. All H atoms, apart from the O—H hydrogen involved in the intra­molecular hydrogen bonding with the imine N atom were positioned geometrically and refined using a riding model. The H atoms involved in the intra­molecular hydrogen bond were located in the Fourier difference map wherever feasible. In **2** and **3**, one of the *tert*-butyl groups was disordered [apart from at 120 (2) and 100 (2) K for **2**, and at 100 (2) K for **3**], the sum of the occupancies of the disordered parts was set to equal 1. The data for **2** at 300 (2), 250 (2) and 200 (2) K drop off at high angle, presumably due to the presence of the disorder in the *tert*-butyl group; as a consequence, the data were cut at resolution limits of 0.95, 0.91 and 0.89 Å, respectively. Likewise the data for **3** were also weak at 300 (2) K and were consequently cut at a resolution limit of 0.86 Å. The inter­planar dihedral angle and fold angles were calculated in *OLEX2* (Dolomanov *et al.*, 2012) by measuring the angles between planes com­puted through the six non-H atoms of the two rings. For the acentric structure of **1B** at 120 K, the diffraction data did not establish the absolute structure.

### Raman   

Irradiation was carried out using two UV LED sources (λ ∼ 365 nm) in the dark to minimize conversion back to the ground state and measurements were recorded with the 764 nm laser on a Horiba Jobin Yvon LabRAM HR Raman spectrometer.

### Diffuse reflectance spectroscopy   

The sample was ground to give uniform particle distribution and placed in a 40 × 10 × 2 mm quartz cuvette to ensure optical thickness. A cuvette sample holder with a white polytetra­fluoro­ethyl­ene (PTFE) block spacer was used to load the sample into an Oxford Instruments Cryostat. The sample was irradiated with an Ocean Optics halogen light source and an Avantes AvaSpec-2048-2 CCD detector (placed at an acute angle to minimize detection of specular reflectance) collected the reflectance spectra, which were recorded using *AvaSoft* basic software. Cryostat temperature control was performed using an Oxford Intelligent Temperature Controller and each temperature was stabilized for 10 min or until ±0.1 K before recording the spectrum. A white PTFE block was used to record a reference spectrum before each data set collection. Irradiation was carried out using a 405 nm laser po­inter or UV LEDs after an initial ground-state spectrum was collected. The diffuse reflectance spectra are illustrated as percent reflectance *versus* wavelength and Kubelka–Munk function, *F*(*R*), *versus* wavelength. If *S* is independent of λ, then *F*(*R*) *versus* λ is equivalent to the absorption spectrum for a diffuse reflector. To allow basic trends to be easily observed, moving averages were applied to data during analysis.

## Results and discussion   

### Structural characterization   

Compound **1** was found to produce two different polymorphs upon recrystallization, **1A** and **1B**, which had different morphologies and structures. Polymorph **1A** formed yellow rectangular block-like crystals and crystallized in the triclinic space group *P* 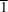, while **1B** formed bright-yellow octa­hedral-shaped crystals and crystallized in the ortho­rhom­bic space group *Pna*2_1_. Only one polymorph was identified during these studies for **2** and **3**, both of which were yellow.

**Figure 2:**
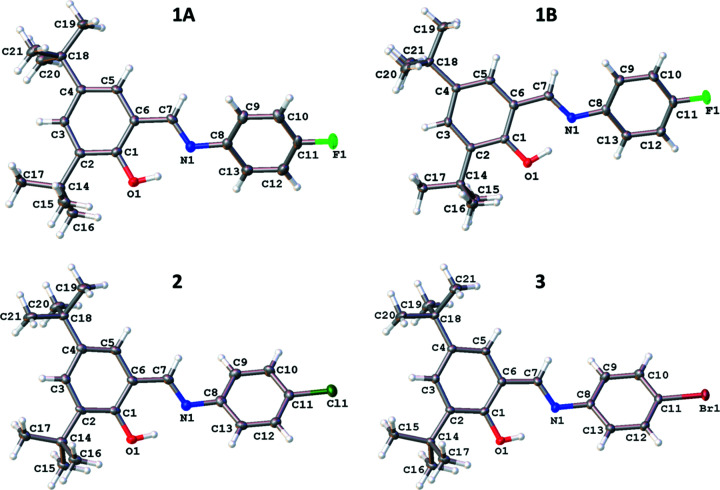
Illustration of the structures of **1A** [at 100 (2) K], **1B** [120 (2) K], **2** [100 (2) K] and **3** [100 (2) K], with the atomic numbering schemes depicted. Displacement ellipsoids are drawn at the 50% probability level.

The four structures are all similar in that they have the same basic backbone with a phenyl group substituted with a hy­droxy and two *tert*-butyl groups, and joined to a halogen-substituted phenyl group *via* an imine group (Fig. 2 ▸). The structures all exist in the enol form at low temperature rather than the less common keto form, with the C7=N1 bond lengths ranging from 1.279 (3) to 1.286 (2) Å and the C1—O1 bond lengths ranging from 1.353 (3) to 1.358 (2) Å, which are consistent with double C=N (typically ∼1.279 Å) and single C—O (typically ∼1.362 Å) bonds, respectively (Allen *et al.*, 1987 ▸). In all cases, the H atom was also located in the Fourier difference map in the vicinity of the O atom, supporting the presence of the enol form of the anil. All the structures contain an intra­molecular O1—H1⋯N1 hydrogen bond with similar parameters, *e.g.* O1⋯N1 distances ranging from 2.544 (2) to 2.633 (3) Å (see Table 2 ▸). The structures also contain weaker inter­molecular C—H⋯O inter­actions (see Table 3 ▸).

**Figure 3:**
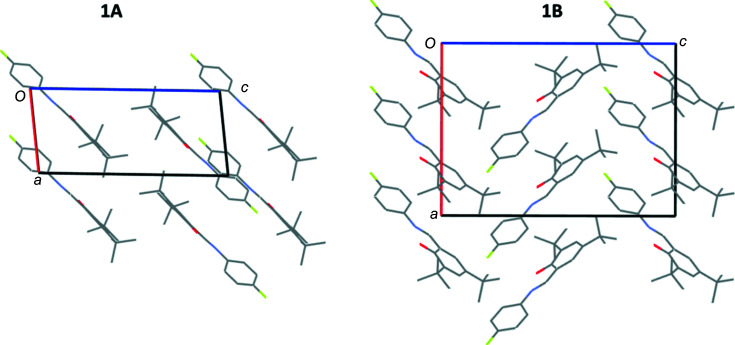
Illustration of the packing of **1A** at 100 (2) K and **1B** at 120 (2) K, looking down the *b* axis. H atoms have been omitted for clarity.

**Figure 4:**
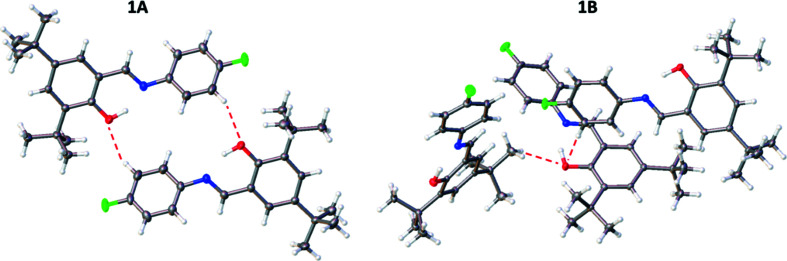
Inter­molecular hydrogen bonding (dashed lines) in **1A** at 100 (2) K and **1B** at 120 (2) K.

The structure of **1A** consists of mol­ecules oriented such that the plane of the mol­ecules is in approximately the (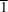
01) plane, with short aromatic C—H⋯O contacts between pairs of adjacent mol­ecules. The *tert*-butyl groups within these pairs are at opposite ends to each other (Fig. 3 ▸). Examining the structure of **1B** shows that the mol­ecules are packed in a com­pletely different manner to **1A**; in **1B**, alternate mol­ecules in the *c*-axis direction are orientated in either the [101] or [10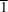] direction (Fig. 3 ▸). Inter­molecular inter­actions in this case are (i) short C—H⋯O contacts involving the H atoms on a methyl group and an aromatic H atom, and (ii) C—H⋯F contacts involving methyl-group H atoms (see Fig. 4 ▸ and Table 3 ▸). The structures of **2** and **3** were found to be iso­structural, crystallizing in the monoclinic space group *P*2_1_/*c*. All of the mol­ecules are oriented such that the plane of the mol­ecules is in approximately the (101) plane, with short aromatic C—H⋯O contacts between pairs of adjacent mol­ecules. Within these pairs, the mol­ecules are arranged such that the *tert*-butyl groups are at opposite ends to each other (Fig. S1). Although in a different crystal system and space group, the structures of **2** and **3** are similar to that of **1A** in terms of the packing and inter­molecular inter­actions.

### Thermal behaviour   

The structures of **2** and **3** are isostructural and upon cooling both undergo a phase transition somewhere between 150 and 120 K, during which the *a*-axis length decreases by ∼0.37 Å for **2** and by ∼0.27 Å for **3**, while the *b* axis increases by ∼0.20 Å for **2** and by ∼0.10 Å for **3**. These changes are accom­panied by a decrease in the β angle of just over 1° in both cases (see Figs. S2 and S3 in the supporting information). Across the full temperature range measured, the behaviour of the unit-cell parameters is slightly different for the two com­pounds but shows many similarities. For **2**, the *a* axis decreases almost linearly until 150 K and then shows a sharp decrease after the phase transition; the *b* axis decreases approximately linearly until 200 K, increases slightly to 150 K and then increases sharply by 120 K; the β angle decreases approximately linearly until 150 K, then shows a sharp decrease to 120 K before increasing slightly at 100 K; and the *c* axis and unit-cell volume decrease almost linearly throughout, with slight inflections at around 175 and 150 K. For **3** overall across the temperature range, upon cooling, the *a* and *c* cell-axes lengths, β angle and unit-cell volume decrease approximately linearly prior to the phase transition and continue to decrease after the phase transition. In the case of the *b* axis, it initially decreases until ∼200 K, increases slightly at 150 K and then increases sharply through the phase transition.

Examining the crystal structures above and below the transition, the cause of the phase transition appears to be dynamic disorder in one of the *tert*-butyl groups; at higher temperature, the group is disordered, while at low temperature, the disorder resolves. In the case of **2**, the disordered *tert*-butyl group is modelled over three positions at 150 (2) K, but is fully ordered at 120 (2) K, while for **3** at 150 (2) K, the *tert*-butyl group is also modelled over three positions, at 120 (2) K it was modelled over two positions and it was only at 100 (2) K that it was fully ordered.

**Figure 5:**
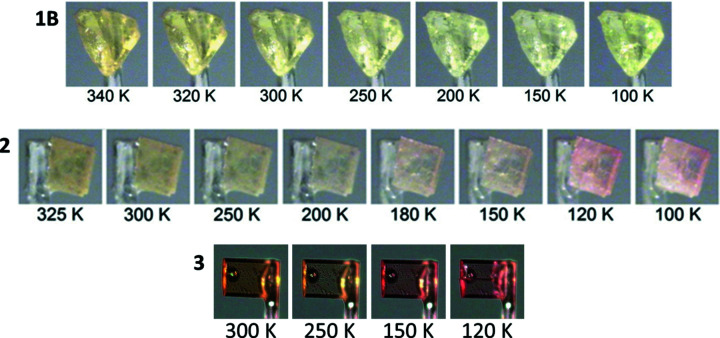
Illustration of the colour change of **1B**, **2** and **3** upon cooling.

The majority of the *N*-salicylideneanilines show thermochromism upon cooling, with com­pounds that are red/orange at room temperature becoming paler or yellow and those that are yellow at room temperature becoming paler. However, it was inter­esting to note that upon cooling, the crystals of **2** and **3** showed an apparent ‘reverse thermochromism’ around the phase-transition temperature, with the crystals becoming more red (Fig. 5 ▸ and Fig. S4 in the supporting information). Diffuse reflectance spectra were also collected for all of the com­pounds and are available in the supporting information (Figs. S5 and S6). No account was taken of the potential effect of fluorescence, which can affect the observed colour upon cooling (Harada *et al.*, 2007 ▸); however, the spectra are presented to support the visually observed trends. In the case of the reflectance spectra for **1**, which is likely to be a mixture of both polymorphs, the shoulder shifts to lower wavelengths upon cooling suggesting a lightening in colour. In the cases of **2** and **3**, there is also a shift in the position of the main shoulder to lower wavelengths upon cooling, but this is also accom­panied by additional changes in the spectra. The spectrum for **2** shows additional changes in the ∼500–580 nm region with additional shoulders appearing. These appear to start by around 200 K and become more pronounced upon further cooling, which is consistent with results observed visually in Fig. 5 ▸. A similar situation, although less pronounced, is observed for **3**, where additional shoulders appear in the range ∼500–580 nm for the spectra at 200 K and below. The apparent ‘reverse thermochromism’ is believed to be related to the phase transition that has occurred rather than the normal thermochromism seen in *N*-salicylideneanilines. It was noted that the dihedral angle between the two six-membered rings decreases by around 1.2–1.9° as the temperature is reduced and in the case of **2**, there is a noticeably larger step around the phase transition between 150 (2) and 120 (2) K. In both cases, the fold angle increases as the temperature is reduced and there is a large step increase of ∼2.3–2.5° between 150 (2) and 120 (2) K (see Table 4 ▸). Although relatively small, it is possible that these structural changes may be related to the observed colour change, as structures with smaller dihedral angles have reduced overlap between the N-atom lone pair and the aromatic aniline, allowing for a stronger O—H⋯N hydrogen bond favoured by the strongly thermochromic com­pounds (Hadjoudis & Mavridis, 2004 ▸; Robert *et al.*, 2009 ▸). In the case of *N*-salicylideneaniline, a similar reverse thermochromism has been seen, whereupon heating above 306 K the colour changes from red to yellow. This was associated with the planar β-form transitioning to the nonplanar disordered α_1_-form; however, the change in structure in this case was much more significant, with a change in the dihedral angle from (β) ∼2° to (α_1_) ∼49° (Arod *et al.*, 2007 ▸). More examples and further studies would be required to confirm a correlation between the colour change observed here and the phase transition having occurred.

### Photochromism   

**Figure 6:**
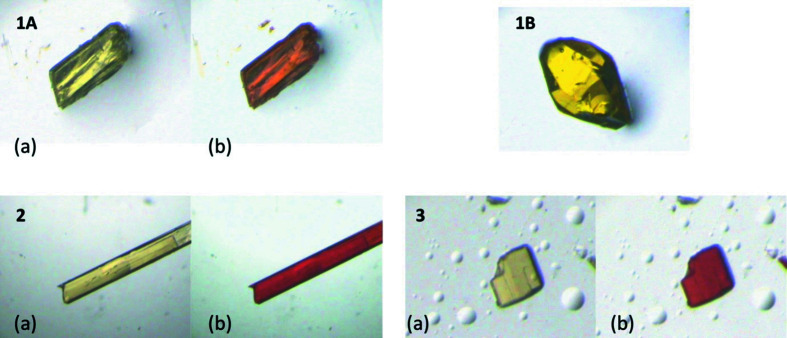
Illustration of the behaviour of each of the crystal structures at room temperature upon irradiation with a UV LED (λ ∼ 365 nm) for (*a*) unirradiated and (*b*) irradiated.

Upon irradiation, three of the crystals (**1A**, **2** and **3**) were found to display photochromism, becoming much darker in colour when irradiated with UV light. On the other hand, polymorph **1B** did not show a colour change even upon pro­longed irradiation (Fig. 6 ▸). The occurrence of photochromism for **3** had been reported previously (Johmoto *et al.*, 2012 ▸).

**Figure 7:**
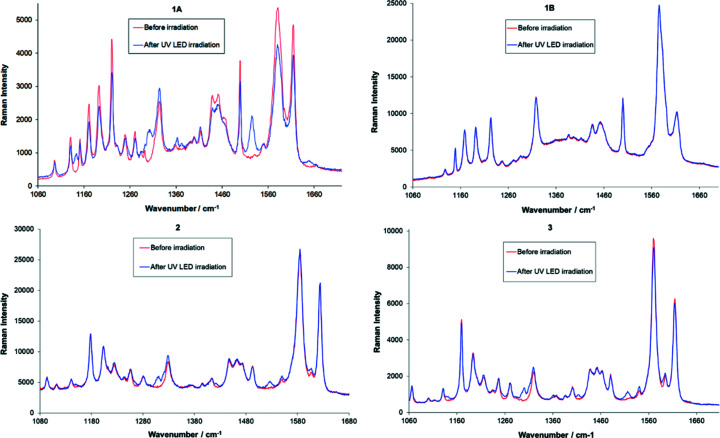
Raman spectra of **1A**, **1B**, **2** and **3** before irradiation (red) and after irradiation (blue) with UV LEDs.

Raman data were collected before irradiation and after irradiation with a UV LED (Fig. 7 ▸). The three crystals displaying photochromism (**1A**, **2** and **3**) all showed the appearance of new peaks upon irradiation; the main peak positions are given in Table 5 ▸. As expected, the spectrum of **1B** showed no change in the Raman spectra upon irradiation. It is clear that only a small amount of the photoproduct has been formed, which is not uncommon as photoyield is often low, particularly without two-photon excitation, and the change is often restricted to the surface of the crystal (Harada *et al.*, 2008 ▸). Therefore, it is unsurprising that even after irradiation with a UV laser, no changes were observed in the single-crystal X-ray structures. Diffuse reflectance spectra before and after irradiation for samples of **2** and **3** are presented in the supporting information (Fig. S7); these support the observation by eye and from the Raman with significant changes in the spectra upon irradiation. In both cases, the position of the shoulder in the reflectance spectra shifts to higher wavelengths upon irradiation. No diffuse reflectance spectra upon irradiation are presented for **1** due to the sample likely being a mixture of polymoprhs.

It has also been found that com­pounds with more ‘space’ in the crystal lattice are more likely to show photochromism as it is easier for the com­pound to undergo the necessary *cis* to *trans* isomerism. The presence of bulky *tert*-butyl groups can help create space and potentially enable photochromism to be displayed (Johmoto *et al.*, 2012 ▸). However, only three of the structures reported herein (**1A**, **2** and **3**) display photochromism. The packing and inter­molecular inter­actions in **1A**, **2** and **3** are relatively similar, as discussed earlier; however, **1B** has significantly different packing and inter­molecular inter­actions. It seems reasonable to suggest that these differences may well link to the different photochromic behaviour of the com­pounds. A link has been proposed between the inter­planar or dihedral angle between the two aromatic rings and the likelihood of this type of com­pound showing photochromism with the observation that photochromic com­pounds tend to have a larger dihedral angle, typically dihedral angles below 20° are associated with nonphotochromic com­pounds, those above 30° are more likely to be photochromic and those between 20 and 30° can display photochromism (Johmoto *et al.*, 2012 ▸). The structures reported here fit in with these general observations and those with the larger dihedral angles between the two aromatic rings are the ones displaying photochromism (see Table 4 ▸). It is possible that in order to form the aromatic C—H⋯O inter­action present in **1A**, **2** and **3**, a larger dihedral angle is required between the two rings, while the C—H⋯O inter­actions in **1B** do not require this twisting, hence photochromism may be favoured for **1A**, **2** and **3** with the larger dihedral angle but is not seen for **1B** due to the smaller dihedral angle.

## Conclusions   

The structures of three related Schiff base com­pounds are reported; for one, (*E*)-2,4-di-*tert*-butyl-6-{[(4-fluoro­phen­yl)imino]­meth­yl}phenol, **1**, two polymorphic structures are reported. The basic structures of the com­pounds are very similar, all existing in the enol form and showing an inter­molecular O—H⋯N hydrogen bond and short C—H⋯O contacts. However, the packing of the two polymorphs of **1** were found to be significantly different. Compounds **2** and **3** were found to be isostructural with each other and displayed a temperature-induced phase transition upon cooling, this was attributed to the disorder in one of the *tert*-butyl groups resolving at low temperature, which was linked to a colour change from yellow to red around the phase transition. Three of the structures, *i.e.* **1A**, **2** and **3**, were found to show photochromism upon irradiation with UV LEDs, while **1B** did not; this was linked with differences in the packing and to the inter­planar or dihedral angle between the two aromatic rings being greater than 25° for the photochromic structures and being less than 25° for **1B**. The presence of photochromism was identified both by eye and Raman spectroscopy.

## Supplementary Material

Crystal structure: contains datablock(s) 1A_100K, 1B_120K, 2_300K, 2_250K, 2_200K, 2_150K, 2_120K, 2_100K, 3_300K, 3_250K, 3_200K, 3_150K, 3_120K, 3_100K, global. DOI: 10.1107/S2053229621008780/wv3002sup1.cif

Structure factors: contains datablock(s) 1A_100K. DOI: 10.1107/S2053229621008780/wv30021A_100Ksup2.hkl

Structure factors: contains datablock(s) 1B_120K. DOI: 10.1107/S2053229621008780/wv30021B_120Ksup3.hkl

Structure factors: contains datablock(s) 2_300K. DOI: 10.1107/S2053229621008780/wv30022_300Ksup4.hkl

Structure factors: contains datablock(s) 2_250K. DOI: 10.1107/S2053229621008780/wv30022_250Ksup5.hkl

Structure factors: contains datablock(s) 2_200K. DOI: 10.1107/S2053229621008780/wv30022_200Ksup6.hkl

Structure factors: contains datablock(s) 2_150K. DOI: 10.1107/S2053229621008780/wv30022_150Ksup7.hkl

Structure factors: contains datablock(s) 2_120K. DOI: 10.1107/S2053229621008780/wv30022_120Ksup8.hkl

Structure factors: contains datablock(s) 2_100K. DOI: 10.1107/S2053229621008780/wv30022_100Ksup9.hkl

Structure factors: contains datablock(s) 3_300K. DOI: 10.1107/S2053229621008780/wv30023_300Ksup10.hkl

Structure factors: contains datablock(s) 3_250K. DOI: 10.1107/S2053229621008780/wv30023_250Ksup11.hkl

Structure factors: contains datablock(s) 3_200K. DOI: 10.1107/S2053229621008780/wv30023_200Ksup12.hkl

Structure factors: contains datablock(s) 3_150K. DOI: 10.1107/S2053229621008780/wv30023_150Ksup13.hkl

Structure factors: contains datablock(s) 3_120K. DOI: 10.1107/S2053229621008780/wv30023_120Ksup14.hkl

Structure factors: contains datablock(s) 3_100K. DOI: 10.1107/S2053229621008780/wv30023_100Ksup15.hkl

Supporting information file. DOI: 10.1107/S2053229621008780/wv30021A_100Ksup16.cml

Supporting information file. DOI: 10.1107/S2053229621008780/wv30022_100Ksup17.cml

Supporting information file. DOI: 10.1107/S2053229621008780/wv30023_100Ksup18.cml

Characterization details, crystal data and additional figures. DOI: 10.1107/S2053229621008780/wv3002sup19.pdf

CCDC references: 2099385, 2099384, 2099383, 2099382, 2099381, 2099380, 2099379, 2099378, 2099377, 2099376, 2099375, 2099374, 2099373, 2099372

## Figures and Tables

**Table 1:** Experimental details

	**1A** at 100 K	**1B** at 120 K	**2** at 100 K	**3** at 100 K
Crystal data				
Chemical formula	C_21_H_26_FNO	C_21_H_26_FNO	C_21_H_26_ClNO	C_21_H_26_BrNO
*M*_r_	327.43	327.43	343.88	388.34
Crystal system, space group	Triclinic, *P*\overline{1}	Orthorhombic, *P* *n* *a*2_1_	Monoclinic, *P*2_1_/*c*	Monoclinic, *P*2_1_/*c*
Temperature (K)	100	120	100	100
*a*, *b*, *c* (Å)	6.5324 (3), 10.6141 (8), 14.1675 (9)	12.2569 (3), 8.9658 (2), 16.5739 (4)	17.3011 (11), 10.6780 (7), 10.1200 (6)	17.4450 (3), 10.69412 (16), 10.15010 (17)
α, β, γ (°)	80.364 (5), 81.094 (4), 74.507 (5)	90, 90, 90	90, 90.252 (6), 90	90, 90.1557 (16), 90
*V* (Å^3^)	926.97 (10)	1821.35 (7)	1869.6 (2)	1893.58 (5)
*Z*	2	4	4	4
Radiation type	Mo *K*α	Mo *K*α	Mo *K*α	Mo *K*α
μ (mm^−1^)	0.08	0.08	0.21	2.18
Crystal size (mm)	0.38 × 0.36 × 0.26	0.46 × 0.43 × 0.10	0.35 × 0.31 × 0.10	0.3 × 0.05 × 0.05

Data collection				
Diffractometer	Oxford Diffraction Xcalibur Sapphire3 Gemini ultra	Oxford Diffraction Xcalibur Sapphire3 Gemini ultra	Oxford Diffraction Xcalibur Sapphire3 Gemini ultra	Agilent SuperNova Dual Source diffractometer with an Atlas detector
Absorption correction	Multi-scan (*CrysAlis PRO*; Oxford Diffraction, 2010 ▸)	Analytical (*CrysAlis PRO*; Oxford Diffraction, 2010 ▸)	Multi-scan (*CrysAlis PRO*; Rigaku OD, 2018 ▸)	Multi-scan (*CrysAlis PRO*; Agilent, 2012 ▸)
*T*_min_, *T*_max_	0.870, 1.000	0.973, 0.992	0.435, 1.000	0.692, 1.000
No. of measured, independent and observed [*I* > 2σ(*I*)] reflections	7178, 3792, 2765	25510, 3712, 3517	13865, 3830, 2740	28200, 4491, 3799
*R*_int_	0.038	0.049	0.087	0.036
(sin θ/λ)_max_ (Å^−1^)	0.625	0.625	0.625	0.658

Refinement				
*R*[*F*^2^ > 2σ(*F* ^2^)], *wR*(*F* ^2^), *S*	0.054, 0.110, 1.04	0.034, 0.077, 1.05	0.071, 0.176, 1.08	0.026, 0.060, 1.04
No. of reflections	3792	3712	3830	4491
No. of parameters	227	226	227	227
No. of restraints	0	1	0	22
H-atom treatment	H atoms treated by a mixture of independent and constrained refinement	H atoms treated by a mixture of independent and constrained refinement	H atoms treated by a mixture of independent and constrained refinement	H atoms treated by a mixture of independent and constrained refinement
Δρ_max_, Δρ_min_ (e Å^−3^)	0.28, −0.20	0.16, −0.16	0.78, −0.33	0.46, −0.22

Computer programs: *CrysAlis PRO* (Oxford Diffraction, 2010 ▸; Rigaku OD, 2018 ▸; Agilent, 2012 ▸), *SHELXS* (Sheldrick, 2008 ▸), *olex2.solve* (Bourhis *et al.*, 2015 ▸), *SHELXL2018* (Sheldrick, 2015 ▸) and *OLEX2* (Dolomanov *et al.*, 2009 ▸).

**Table 2:** O—H⋯N hydrogen-bond geometry (Å, °)

	* T* (K)	*D*—H⋯*A*	*D*—H	H⋯*A*	*D*⋯*A*	*D*—H⋯*A*
**1A**	100	O1—H1⋯N1	0.94 (3)	1.72 (3)	2.587 (2)	151 (2)
**1B**	120	O1—H1⋯N1	0.96 (3)	1.64 (3)	2.544 (2)	155 (2)
**2**	300	O1—H1⋯N1	0.84 (4)	1.84 (4)	2.612 (4)	151 (4)
	250	O1—H1⋯N1	0.91 (3)	1.76 (4)	2.615 (4)	155 (3)
	200	O1—H1⋯N1	0.90 (3)	1.78 (3)	2.611 (3)	153 (3)
	150	O1—H1⋯N1	0.92 (3)	1.77 (3)	2.615 (3)	151 (3)
	120	O1—H1⋯N1	0.86 (4)	1.82 (4)	2.633 (3)	157 (4)
	100	O1—H1⋯N1	0.94 (4)	1.77 (4)	2.626 (3)	150 (3)
**3**	300	O1—H1⋯N1	0.83 (5)	1.84 (5)	2.614 (4)	154 (5)
	250	O1—H1⋯N1	0.84 (3)	1.83 (3)	2.612 (3)	154 (3)
	200	O1—H1⋯N1	0.82 (3)	1.85 (3)	2.611 (2)	153 (3)
	150	O1—H1⋯N1	0.86 (1)	1.83 (2)	2.612 (2)	152 (3)
	120	O1—H1⋯N1	0.86 (1)	1.84 (2)	2.622 (2)	151 (3)
	100	O1—H1⋯N1	0.85 (1)	1.84 (1)	2.6257 (18)	152 (2)

**Table 3:** C—H⋯O and C—H⋯F hydrogen-bond geometry (Å, °)

	*T* (K)	*D*—H⋯*A*	*D*—H	H⋯*A*	*D*⋯*A*	*D*—H⋯*A*
**1A**	100	C12—H12⋯O1^i^	0.95	2.62	3.345 (2)	133
**1B**	120	C10—H10⋯O1^ii^	0.95	2.60	3.523 (3)	165
		C19—H19*B*⋯O1^iii^	0.98	2.72	3.522 (3)	140
		C17—H17*A*⋯F1^iv^	0.98	2.57	3.453 (3)	150
**2**	300	C12—H12⋯O1^v^	0.93	2.71	3.461 (4)	138
	250	C12—H12⋯O1^v^	0.94	2.68	3.438 (3)	138
	200	C12—H12⋯O1^v^	0.95	2.65	3.415 (2)	138
	150	C12—H12⋯O1^vi^	0.95	2.56	3.359 (3)	142
	100	C12—H12⋯O1^vi^	0.95	2.54	3.343 (4)	142
	150	C12—H12⋯O1^v^	0.95	2.63	3.396 (3)	138
	120	C12—H12⋯O1^vi^	0.95	2.56	3.359 (3)	142
**3**	300	C12—H12⋯O1^vii^	0.93	2.76	3.522 (3)	140
	250	C12—H12⋯O1^vii^	0.94	2.73	3.500 (2)	140
	200	C12—H12⋯O1^vii^	0.94	2.71	3.483 (3)	140
	150	C12—H12⋯O1^vii^	0.95	2.67	3.458 (2)	140
	120	C12—H12⋯O1^vii^	0.95	2.62	3.425 (2)	142
	100	C12—H12⋯O1^vii^	0.95	2.61	3.415 (2)	143

Symmetry codes: (i) −*x*, −*y* + 2, −*z*; (ii) *x* + {1\over 2}, −*y* + {3\over 2}, *z*; (iii) −*x* + {3\over 2}, *y* + {1\over 2}, *z* + {1\over 2}; (iv) −*x* + 2, −*y* + 1, *z* + {1\over 2}; (v) −*x* + 2, −*y* + 1, −*z* + 1; (vi) −*x* + 2, −*y*, −*z* + 1; (vii) −*x*, −*y*, −*z*.

**Table 4:** Dihedral angles (°) between planes calculated through the six atoms of the two rings

	*T* (K)	Dihedral angle (°)	Fold angle (°)
**1A**	100	39.03 (5)	8.68 (5)
**1B**	120	20.61 (7)	3.24 (7)
**2**	300	26.75 (7)	9.01 (9)
	250	26.56 (8)	8.87 (8)
	200	26.33 (6)	9.09 (6)
	150	25.80 (9)	9.28 (9)
	120	24.96 (10)	11.84 (10)
	100	24.83 (9)	12.05 (9)
**3**	300	25.83 (8)	9.29 (9)
	250	25.49 (6)	9.45 (6)
	200	25.33 (8)	9.69 (7)
	150	24.88 (7)	10.16 (7)
	120	24.70 (7)	12.49 (7)
	100	24.63 (5)	13.20 (5)

**Table 5:** Position of main peaks that appear in Raman upon irradiation

Compound	New peaks (cm^−1^)
**1A**	1651, 1525, 1373, 1302 and 1143
**1B**	–
**2**	1528, 1423, 1311 and 1152
**3**	1518, 1418, 1301 and 1134
