# Supplementary material for: Selected solid-state behaviour of three di-tert-butyl-substituted N-salicylideneaniline derivatives: temperature-induced phase transitions and chromic behaviour
Source: Acta Crystallogr C Struct Chem. 2021 Sep 29;77(Pt 10):659–67. doi: 10.1107/S2053229621008780 (PMC8491094; doi:10.1107/S2053229621008780)
Supplement: Supplementary file 19 [file c-77-00659-sup19.pdf]

STRUCTURAL
CHEMISTRY

Volume 77 (2021)

Supporting information for article:

Selected solid-state behaviour of three di-*tert*-butyl-substituted *N*-salicylideneaniline derivatives: temperature-induced phase transitions and chromic behaviour

Helen E. Mason, Judith A. K. Howard and Hazel A. Sparkes

S1. Characterisation

S1.1. (E)-2,4-di-tert-butyl-6-(((4-fluorophenyl)imino)methyl)phenol, 1

Compound **1** was obtained in a yield of 92% (0.378 g). *Elemental Analysis, actual% (predicted%):* C= 76.39(77.03); H= 7.92 (8.00); N= 4.20 (4.28).

S1.2. (E)-2,4-di-tert-butyl-6-(((4-chlorophenyl)imino)methyl)phenol, 2

Compound **2** was obtained in a yield of 97% (0.837 g). *Elemental analysis, actual % (predicted %):* C = 73.42 (73.35); H = 7.60 (7.62); N = 3.89 (4.07).

S1.3. (E)-2,4-di-tert-butyl-6-(((4-bromophenyl)imino)methyl)phenol, 3

Compound **3** was obtained in a yield of 87% (0.422 g). *Elemental Analysis, actual% (predicted%):* C= 64.84(64.95); H= 6.75(6.75); N= 3.61 (3.61).

Table S1 Further Experimental details

	(2_300K)	(2_250K)	(2_200K)	(2_150K)
Crystal data				
Chemical formula	$C_{21}H_{26}ClNO$	$C_{21}H_{26}ClNO$	$C_{21}H_{26}ClNO$	$C_{21}H_{26}ClNO$
M_r	343.88	343.88	343.88	343.88
Crystal system, space group	Monoclinic, $P2_1/c$	Monoclinic, $P2_1/c$	Monoclinic, $P2_1/c$	Monoclinic, $P2_1/c$
Temperature (K)	300	250	200	150
a, *b*, *c* (Å)	17.9412 (17), 10.5067 (7), 10.3890 (7)	17.8739 (13), 10.4846 (7), 10.3312 (6)	17.8132 (12), 10.4564 (6), 10.2814 (6)	17.7326 (13), 10.4696 (7), 10.2385 (7)
α, β, γ (°)	90, 92.719 (7), 90	90, 92.296 (5), 90	90, 91.965 (5), 90	90, 91.536 (6), 90
V (Å^3)	1956.2 (3)	1934.5 (2)	1913.9 (2)	1900.1 (2)
Z	4	4	4	4
Radiation type	Mo *K*α	Mo *K*α	Mo *K*α	Mo *K*α

μ (mm^{-1})	0.20	0.20	0.21	0.21
Crystal size (mm)	0.33 × 0.28 × 0.10	0.33 × 0.28 × 0.10	0.33 × 0.28 × 0.10	0.33 × 0.28 × 0.10
Data collection				
Diffractometer	Xcalibur, Sapphire3, Gemini ultra	Xcalibur, Sapphire3, Gemini ultra	Xcalibur, Sapphire3, Gemini ultra	Xcalibur, Sapphire3, Gemini ultra
Absorption correction	Analytical *CrysAlis PRO*, Oxford Diffraction Ltd., Version 1.171.34.44 (release 25-10-2010 CrysAlis171 .NET) (compiled Oct 25 2010,18:11:34) Analytical numeric absorption correction using a multifaceted crystal model based on expressions derived by R.C. Clark & J.S. Reid. (Clark, R. C. & Reid, J. S. (1995). Acta Cryst. A51, 887-897)	Multi-scan *CrysAlis PRO*, Oxford Diffraction Ltd., Version 1.171.34.44 (release 25-10-2010 CrysAlis171 .NET) (compiled Oct 25 2010,18:11:34) Analytical numeric absorption correction using a multifaceted crystal model based on expressions derived by R.C. Clark & J.S. Reid. (Clark, R. C. & Reid, J. S. (1995). Acta Cryst. A51, 887-897)	Multi-scan *CrysAlis PRO*, Oxford Diffraction Ltd., Version 1.171.34.44 (release 25-10-2010 CrysAlis171 .NET) (compiled Oct 25 2010,18:11:34) Empirical absorption correction using spherical harmonics, implemented in SCALE3 ABSPACK scaling algorithm.	Multi-scan *CrysAlis PRO*, Agilent Technologies, Version 1.171.35.10 (release 06-04-2011 CrysAlis171 .NET) (compiled Apr 6 2011,09:17:13) Empirical absorption correction using spherical harmonics, implemented in SCALE3 ABSPACK scaling algorithm.
T_{min}, T_{max}	0.947, 0.981	0.651, 1.000	0.947, 1.000	0.795, 1.000
No. of measured, independent and observed [$I > 2\sigma(I)$] reflections	5532, 2382, 1501	5874, 2688, 1737	11973, 2833, 2216	8155, 3481, 2376
R_{int}	0.050	0.048	0.055	0.053
θ_{max} (°)	22.0	23.0	23.5	25.4
$(\sin \theta/\lambda)_{max}$ ($Å^{-1}$)	0.526	0.549	0.562	0.602

Refinement				
$R[F^2 > 2\sigma(F^2)]$, $wR(F^2)$, S	0.056, 0.133, 1.02	0.062, 0.145, 1.06	0.051, 0.120, 1.03	0.063, 0.138, 1.08
No. of reflections	2382	2688	2833	3481
No. of parameters	277	277	277	289
No. of restraints	169	181	163	151
H-atom treatment	H atoms treated by a mixture of independent and constrained refinement	H atoms treated by a mixture of independent and constrained refinement	H atoms treated by a mixture of independent and constrained refinement	H atoms treated by a mixture of independent and constrained refinement
$\Delta\rho_{max}$, $\Delta\rho_{min}$ (e Å^{-3})	0.16, −0.15	0.16, −0.19	0.26, −0.23	0.25, −0.30

	(2_120K)	(3_300K)	(3_250K)	(3_200K)
Crystal data				
Chemical formula	$C_{21}H_{26}ClNO$	$C_{21}H_{26}BrNO$	$C_{21}H_{26}BrNO$	$C_{21}H_{26}BrNO$
M_r	343.88	388.34	388.34	388.34
Crystal system, space group	Monoclinic, $P2_1/c$	Monoclinic, $P2_1/c$	Monoclinic, $P2_1/c$	Monoclinic, $P2_1/c$
Temperature (K)	120	300	250	200
a, *b*, *c* (Å)	17.3623 (8), 10.6691 (4), 10.1512 (6)	18.0356 (7), 10.5891 (3), 10.3641 (3)	17.9642 (4), 10.5593 (2), 10.3153 (2)	17.8944 (4), 10.54749 (18), 10.2693 (2)
α, β, γ (°)	90, 90.123 (5), 90	90, 92.894 (3), 90	90, 92.535 (2), 90	90, 92.1529 (18), 90
V (Å^3)	1880.41 (15)	1976.82 (11)	1954.79 (8)	1936.88 (6)

Z	4	4	4	4
Radiation type	Mo *K*α	Mo *K*α	Mo *K*α	Mo *K*α
μ (mm^{-1})	0.21	2.09	2.11	2.13
Crystal size (mm)	0.35 × 0.31 × 0.10	0.3 × 0.05 × 0.05	0.3 × 0.05 × 0.05	0.3 × 0.05 × 0.05
Data collection				
Diffractometer	Xcalibur, Sapphire3, Gemini ultra	SuperNova, Dual, Cu at zero, Atlas	SuperNova, Dual, Cu at zero, Atlas	SuperNova, Dual, Cu at zero, Atlas
Absorption correction	Multi-scan *CrysAlis PRO* 1.171.39.46 (Rigaku Oxford Diffraction, 2018) Empirical absorption correction using spherical harmonics, implemented in SCALE3 ABSPACK scaling algorithm.	Multi-scan *CrysAlis PRO*, Agilent Technologies, Version 1.171.36.32 (release 02-08-2013 CrysAlis171 .NET) (compiled Aug 2 2013,16:46:58) Empirical absorption correction using spherical harmonics, implemented in SCALE3 ABSPACK scaling algorithm.	Multi-scan *CrysAlis PRO*, Agilent Technologies, Version 1.171.36.32 (release 02-08-2013 CrysAlis171 .NET) (compiled Aug 2 2013,16:46:58) Empirical absorption correction using spherical harmonics, implemented in SCALE3 ABSPACK scaling algorithm.	Multi-scan *CrysAlis PRO*, Agilent Technologies, Version 1.171.36.32 (release 02-08-2013 CrysAlis171 .NET) (compiled Aug 2 2013,16:46:58) Empirical absorption correction using spherical harmonics, implemented in SCALE3 ABSPACK scaling algorithm.
T_{min}, T_{max}	0.846, 1.000	0.744, 1.000	0.690, 1.000	0.660, 1.000
No. of measured, independent and observed [$I > 2\sigma(I)$] reflections	14119, 3860, 2869	13771, 3195, 2444	29546, 4650, 3079	29301, 4612, 3377
R_{int}	0.080	0.036	0.048	0.043
θ_{max} (°)	26.4	24.4	27.9	27.9

$(\sin \theta/\lambda)_{max}$ (Å^{-1})	0.625	0.581	0.658	0.658
Refinement				
$R[F^2 > 2\sigma(F^2)]$, $wR(F^2)$, S	0.072, 0.184, 1.08	0.044, 0.118, 1.05	0.039, 0.101, 1.02	0.035, 0.083, 1.02
No. of reflections	3860	3195	4650	4612
No. of parameters	227	278	278	290
No. of restraints	0	181	181	175
H-atom treatment	H atoms treated by a mixture of independent and constrained refinement	H atoms treated by a mixture of independent and constrained refinement	H atoms treated by a mixture of independent and constrained refinement	H atoms treated by a mixture of independent and constrained refinement
$\Delta\rho_{max}$, $\Delta\rho_{min}$ (e Å^{-3})	0.72, −0.39	0.35, −0.42	0.33, −0.36	0.37, −0.31

	(3_150K)	(3_120K)	(3_100K)
Crystal data			
Chemical formula	$C_{21}H_{26}BrNO$	$C_{21}H_{26}BrNO$	$C_{21}H_{26}BrNO$
M_r	388.34	388.34	388.34
Crystal system, space group	Monoclinic, $P2_1/c$	Monoclinic, $P2_1/c$	Monoclinic, $P2_1/c$
Temperature (K)	150	120	100
a, b, c (Å)	17.8028 (3), 10.55679 (16), 10.21910 (18)	17.5364 (3), 10.65933 (19), 10.1718 (2)	17.4450 (3), 10.69412 (16), 10.15010 (17)
α, β, γ (°)	90, 91.6950 (16), 90	90, 90.6047 (16), 90	90, 90.1557 (16), 90

V (Å^3)	1919.74 (6)	1901.26 (6)	1893.58 (5)
Z	4	4	4
Radiation type	Mo *K*α	Mo *K*α	Mo *K*α
μ (mm^{-1})	2.15	2.17	2.18
Crystal size (mm)	0.3 × 0.05 × 0.05	0.3 × 0.05 × 0.05	0.3 × 0.05 × 0.05
Data collection			
Diffractometer	SuperNova, Dual, Cu at zero, Atlas	SuperNova, Dual, Cu at zero, Atlas	SuperNova, Dual, Cu at zero, Atlas
Absorption correction	Multi-scan *CrysAlis PRO*, Agilent Technologies, Version 1.171.36.32 (release 02-08-2013 CrysAlis171 .NET) (compiled Aug 2 2013,16:46:58) Empirical absorption correction using spherical harmonics, implemented in SCALE3 ABSPACK scaling algorithm.	Multi-scan *CrysAlis PRO*, Agilent Technologies, Version 1.171.36.32 (release 02-08-2013 CrysAlis171 .NET) (compiled Aug 2 2013,16:46:58) Empirical absorption correction using spherical harmonics, implemented in SCALE3 ABSPACK scaling algorithm.	Multi-scan *CrysAlis PRO*, Agilent Technologies, Version 1.171.36.24 (release 03-12-2012 CrysAlis171 .NET) (compiled Dec 3 2012,18:21:49) Empirical absorption correction using spherical harmonics, implemented in SCALE3 ABSPACK scaling algorithm.
T_{min}, T_{max}	0.660, 1.000	0.683, 1.000	0.692, 1.000
No. of measured, independent and observed [$I > 2\sigma(I)$] reflections	28980, 4570, 3604	44888, 4509, 3697	28200, 4491, 3799
R_{int}	0.040	0.045	0.036
θ_{max} (°)	27.9	27.9	27.9
$(\sin \theta/\lambda)_{max}$ (Å^{-1})	0.658	0.658	0.658

Refinement			
$R[F^2 > 2\sigma(F^2)]$, $wR(F^2)$, S	0.031, 0.073, 1.03	0.032, 0.074, 1.05	0.026, 0.060, 1.04
No. of reflections	4570	4509	4491
No. of parameters	258	258	227
No. of restraints	67	103	22
H-atom treatment	H atoms treated by a mixture of independent and constrained refinement	H atoms treated by a mixture of independent and constrained refinement	H atoms treated by a mixture of independent and constrained refinement
$\Delta\rho_{max}$, $\Delta\rho_{min}$ (e Å^{-3})	0.45, −0.36	0.47, −0.42	0.46, −0.22

Computer programs: *CrysAlis PRO*, Oxford Diffraction Ltd., Version 1.171.34.44 (release 25-10-2010 CrysAlis171 .NET) (compiled Oct 25 2010, 18:11:34), Agilent Technologies, Version 1.171.35.3 (release 07-12-2010 CrysAlis171 .NET) (compiled Dec 7 2010, 09:24:43), Version 1.171.35.10 (release 06-04-2011 CrysAlis171 .NET) (compiled Apr 6 2011, 09:17:13), *CrysAlis PRO* 1.171.39.46 (Rigaku OD, 2018), Version 1.171.36.32 (release 02-08-2013 CrysAlis171 .NET) (compiled Aug 2 2013, 16:46:58), Version 1.171.36.24 (release 03-12-2012 CrysAlis171 .NET) (compiled Dec 3 2012, 18:21:49), *SHELXS* (Sheldrick, 2008), olex2.solve 1.3 (Bourhis *et al.*, 2015), *SHELXL* 2018/3 (Sheldrick, 2015), Olex2 1.3 (Dolomanov *et al.*, 2009).

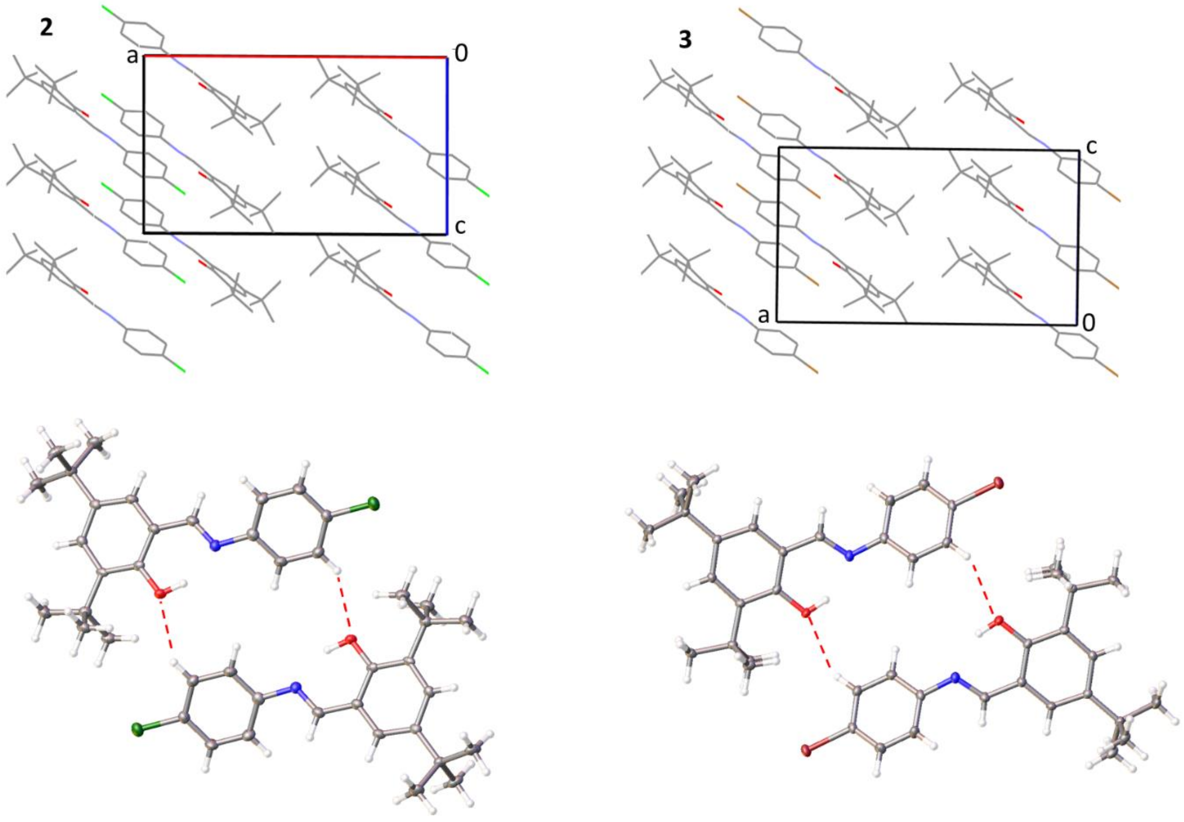

Figure S1 Illustration of (top) the packing of 2 and 3 at 100(2) K looking down the b axis. Hydrogen atoms omitted for clarity. (bottom) C-H···O interactions.

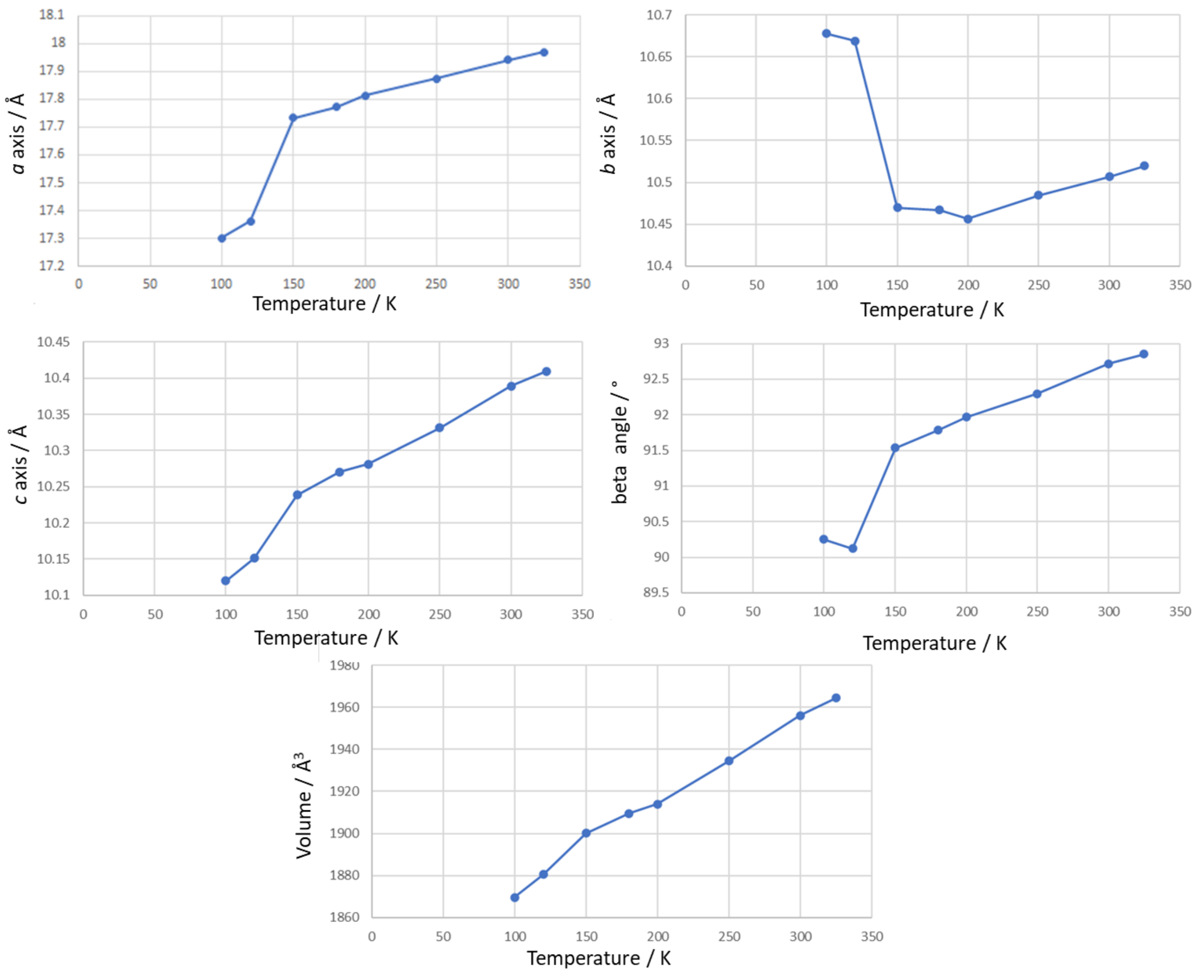

Figure S2 Change in cell parameters for **2** as a function of temperature.

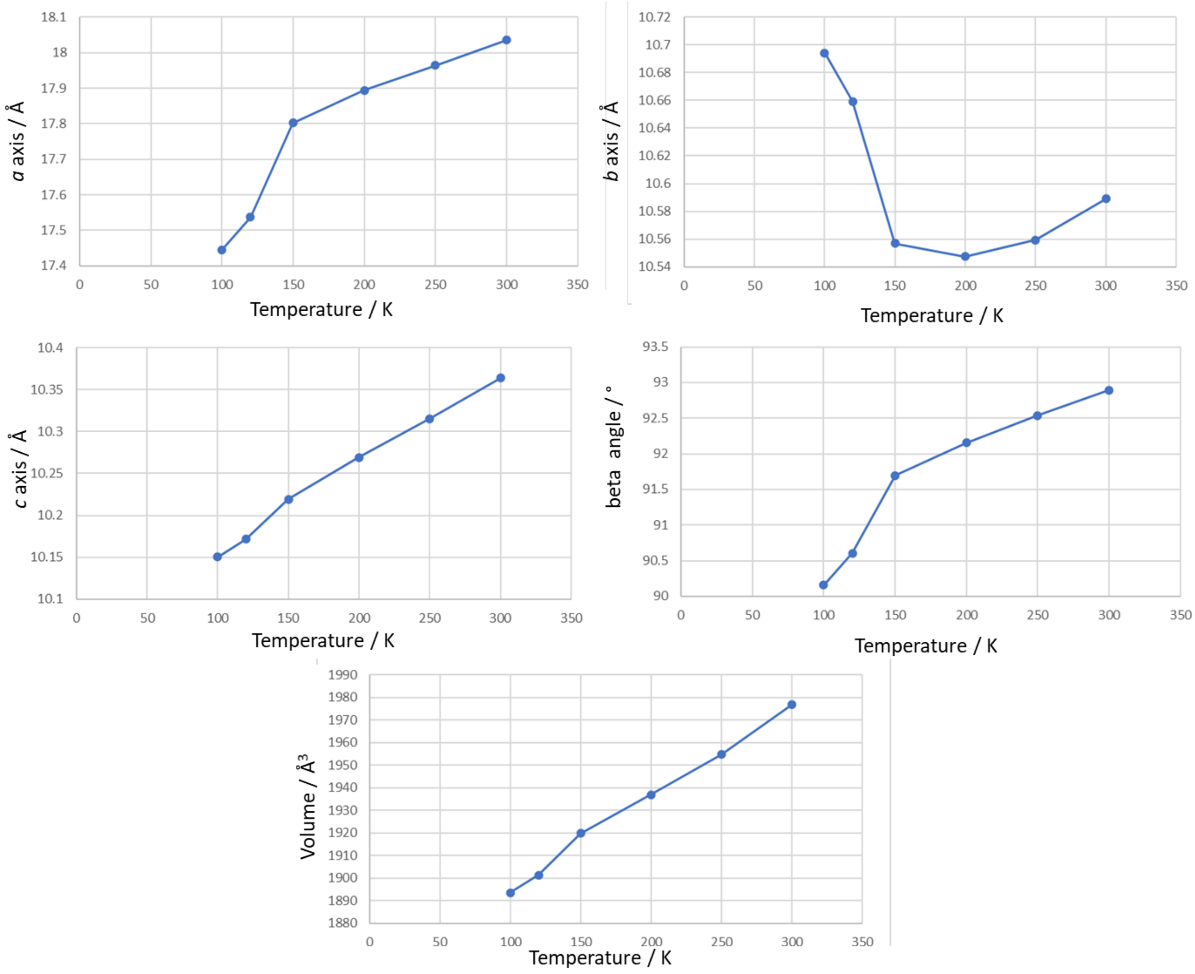

Figure S3 Change in cell parameters for 3 as a function of temperature.

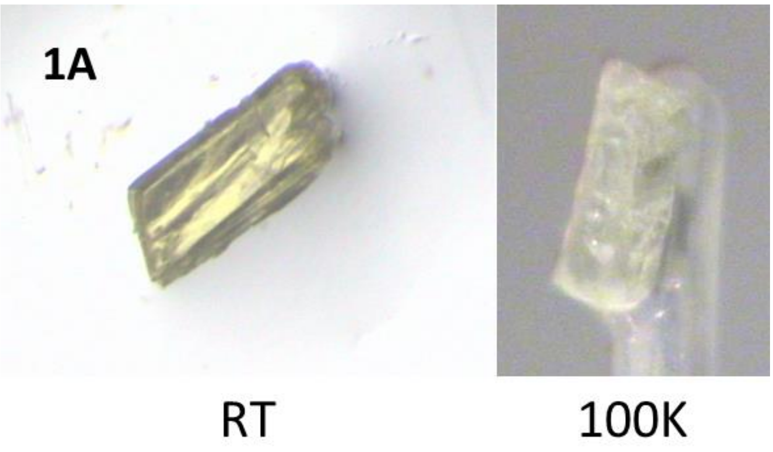

Figure S4 Colour of **1A** at room temperature (RT) and 100(2) K.

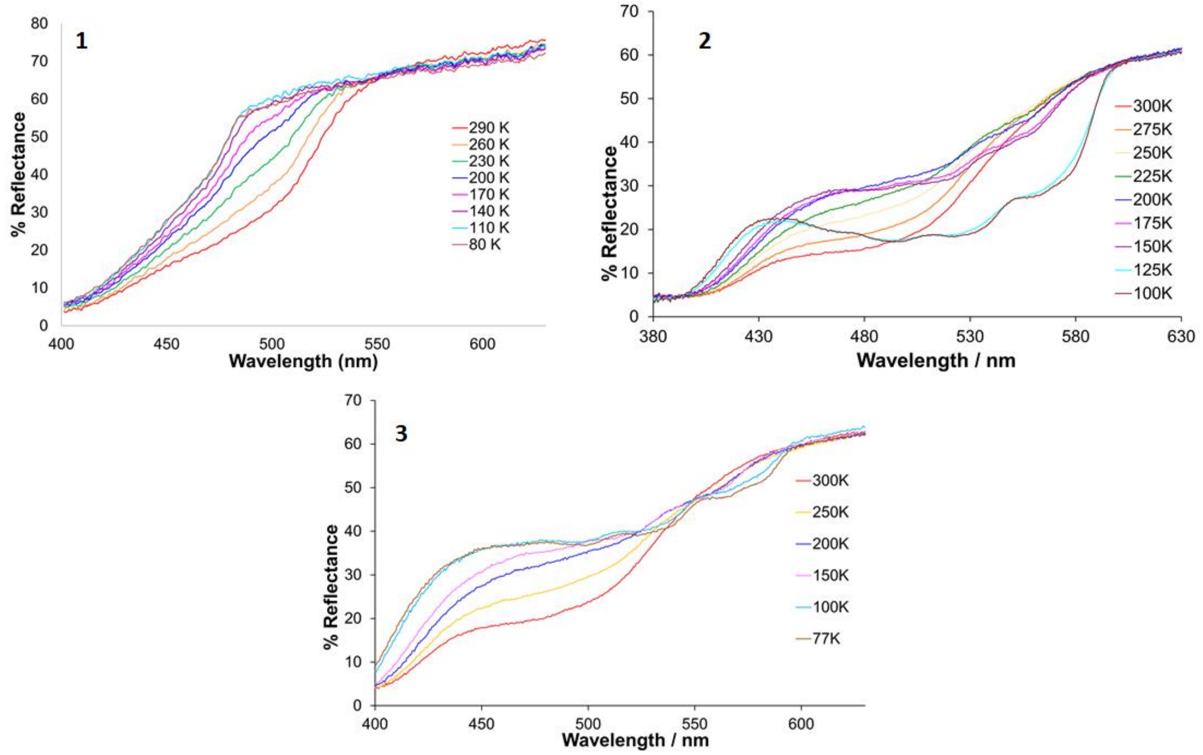

Figure S5 Diffuse reflectance spectra for **1** (likely to be a mixture of both polymorphs), **2** and **3**, displayed as reflectance versus wavelength.

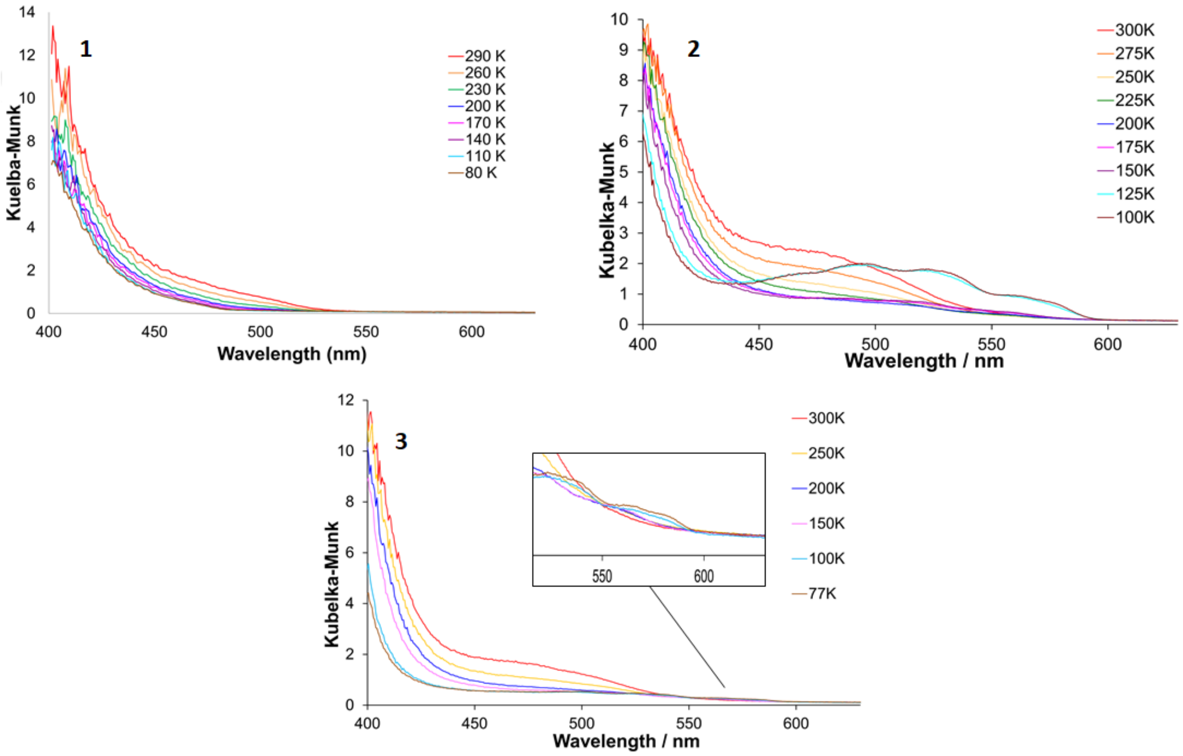

Figure S6 Diffuse reflectance spectra for **1** (likely to be a mixture of both polymorphs), **2** and **3**, displayed as Kubelka-Munk versus wavelength.

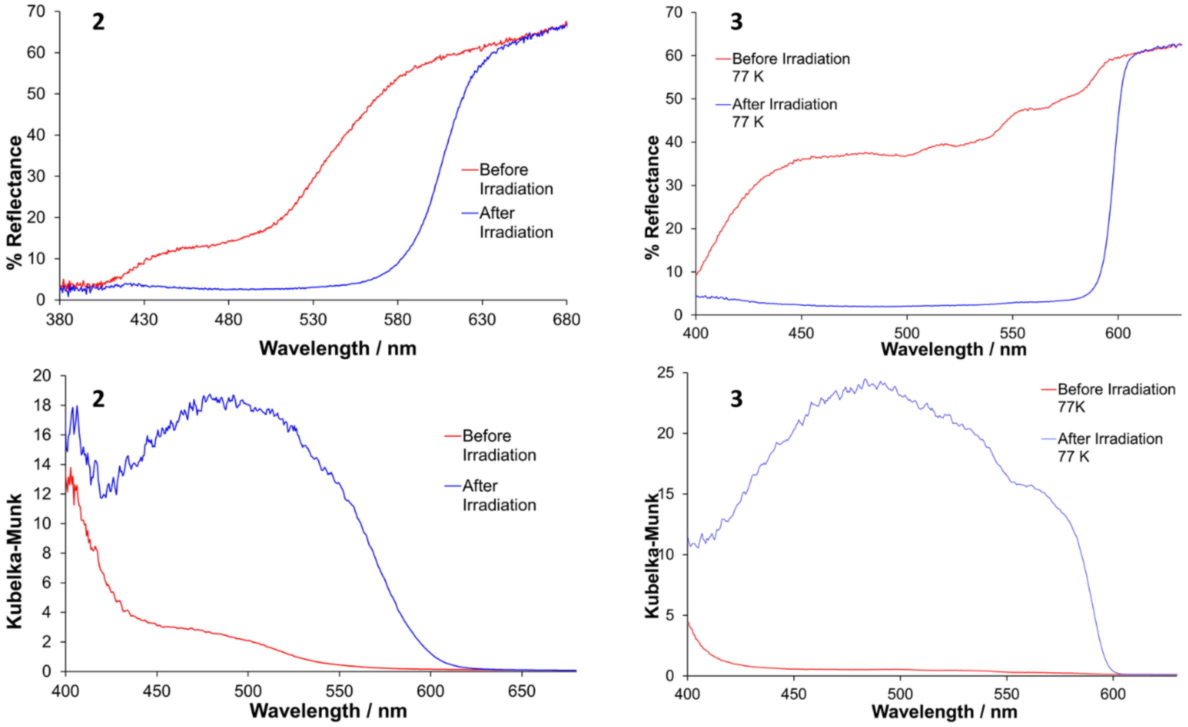

Figure S7 Reflectance spectra for (left) **2** at 298 K and (right) **3** at 77 K before and after irradiation, presented as (top) % reflectance versus wavelength and (bottom) Kubelka-Munk versus wavelength.
